# ChromatoShiny: an interactive R/Shiny App for plotting chromatography profiles

**DOI:** 10.12688/wellcomeopenres.19708.2

**Published:** 2024-01-22

**Authors:** Natalia Y. Kochanova, Maria Alba Abad, Petra Vizjak, A. Arockia Jeyaprakash, William C. Earnshaw, Georg Kustatscher

**Affiliations:** 1 Wellcome Centre for Cell Biology, The University of Edinburgh, Edinburgh, Scotland, EH9 3BF, UK; 2 Biomedical Center Munich, Ludwig-Maximilians-Universitat Munchen, Munich, Planegg-Martinsried, Bavaria, Germany; 3 Gene Center and Department of Biochemistry, Ludwig-Maximilians-Universitat Munchen, Munich, Bavaria, 81377, Germany

**Keywords:** Chromatography, Shiny apps, Äkta machines

## Abstract

**Background:**

Unicorn™ software on Äkta liquid chromatography instruments outputs chromatography profiles of purified biological macromolecules. While the plots generated by the instrument software are very helpful to inspect basic chromatogram properties, they lack a range of useful annotation, customization and export options.

**Methods:**

We use the R Shiny framework to build an interactive app that facilitates the interpretation of chromatograms and the generation of figures for publications.

**Results:**

The app allows users to fit a baseline, to highlight selected fractions and elution volumes inside or under the plot (e.g. those used for downstream biochemical/biophysical/structural analysis) and to zoom into the plot. The app is freely available at https://ChromatoShiny.bio.ed.ac.uk.

**Conclusions:**

It requires no programming experience, so we anticipate that it will enable chromatography users to create informative, annotated chromatogram plots quickly and simply.

## Introduction

Purification of biological macromolecules using Äkta or other manufacturer’s liquid chromatography (LC) instruments is a standard biochemical procedure routinely used in the life sciences ( Curry, 2015; Hillen *et al.*, 2020; Jeyaprakash *et al.*, 2007). Typically, the separation of macromolecules is monitored by UV detectors operating at wavelengths of 260, 280 and often 230 nm. The Äkta machines are equipped with Unicorn ^TM ^software, which can create chromatography plots and export the underlying data (intensity over elution time) as .txt or .csv files or the plots as screenshots or .pdf. Generation of additional features on exported plots or plots generated from the exported data, such as fitting a baseline, zooming in and highlighting particular fractions or elution volume, requires manual selection of the data, working with plots in image editors or even, in some cases, programming. Moreover, for high-throughput plotting of many files, repetitive fine manual work will be required. To overcome these difficulties for users, we built an app using the R shiny framework ( Chang *et al.*, 2021). The app has a simple interface and allows users to build chromatography plots from .txt Unicorn™ files and easily manipulate various features of the plot. In case of data from non-Äkta instruments, it can be entered in the available template and also plotted with the app. The plots can be further exported as .pdf, .eps or .tiff files for use in publications.

## Materials and methods

### Protein expression and purification

Full length Survivin K62A was cloned into a pRSET-His-GFP vector as an N-terminally His-GFP-tagged protein with a 3C cleavage site. The vector was transformed in *E. coli* BL21 Gold strain and grown in Super broth media at 37°C until O.D 0.8. Cultures were induced over night at 18°C with 0.35 mM IPTG. Pelleted cells were lysed in a buffer containing 20 mM Tris–HCl pH 8, 150 mM NaCl, 25 mM imidazole and 2 mM 2-mercaptoethanol. His-GFP-Survivin was purified by affinity chromatography using 5ml of His-Pur Ni-NTA beads (Thermo Fisher). The protein-bound beads were washed with 20 column volumes of lysis buffer followed by 20 column volumes of 20 mM Tris–HCl pH 8, 1M NaCl, 50 mM KCl, 10 mM MgCl _2_, 2 mM ATP, 25 mM imidazole and 2 mM 2-mercaptoethanol. The protein was cleaved on the beads with 0.5 g of 3C protease in lysis buffer over night at 4°C. The cleaved protein was concentrated with a 10 kDa concentrator (Millipore), and the concentrated protein was loaded onto a Superdex 200 increase 10/300 GL column (Cytiva) equilibrated with 20 mM Hepes pH 7.5, 100 mM NaCl and 2 mM DTT.

### App design

The Shiny app is based on a custom R function, which processes the imported .txt file and uses the ggplot2 package ( Wickham, 2016) to create the graphical output. Because of the possibility to easily add different features to a ggplot2 plot, the plotting part of the function was built as a decision tree, encoded as “true” or “false” in each variable, corresponding to a particular decision. In the user interface of the app, these variables are defined by ticking a box - for example, to plot the area under the curve. The dropdown menu used to select particular fractions is generated by a PickerInput() Shiny approach ( Perrier *et al.*, 2022), and the color palettes are achieved using the “colourpicker” package ( Attali, 2021). The other particular packages used in the app are “baseline” for plotting the baseline of the plot ( Liland *et al.*, 2010), “ggrepel” for text labeling ( Slowikowski, 2023), “Cairo” for saving the output of the plot ( Urbanek & Horner, 2022), “png” for working with .png images ( Urbanek, 2022), “readxl” and “writexl” for working with excel files ( Ooms, 2023; Wickham & Bryan, 2023) as well as “shinydashboard” for some elements of the user interface ( Chang & Borges Ribeiro, 2021).

### Software

R version 4.2.0 (2022-04-22) and RStudio 2022.07.2+576 for macOS was used. The versions of the packages used were: ggplot2 3.4.1, shiny 1.7.4, shinyWidgets 0.7.6, shinydashboard 0.7.2, colourpicker 1.2.0, Cairo 1.6-0, baseline 1.3-4, ggrepel 0.9.2.9999, png 0.1-8, readxl 1.4.2, writexl 1.4.2.

## Results

**Figure 1.**
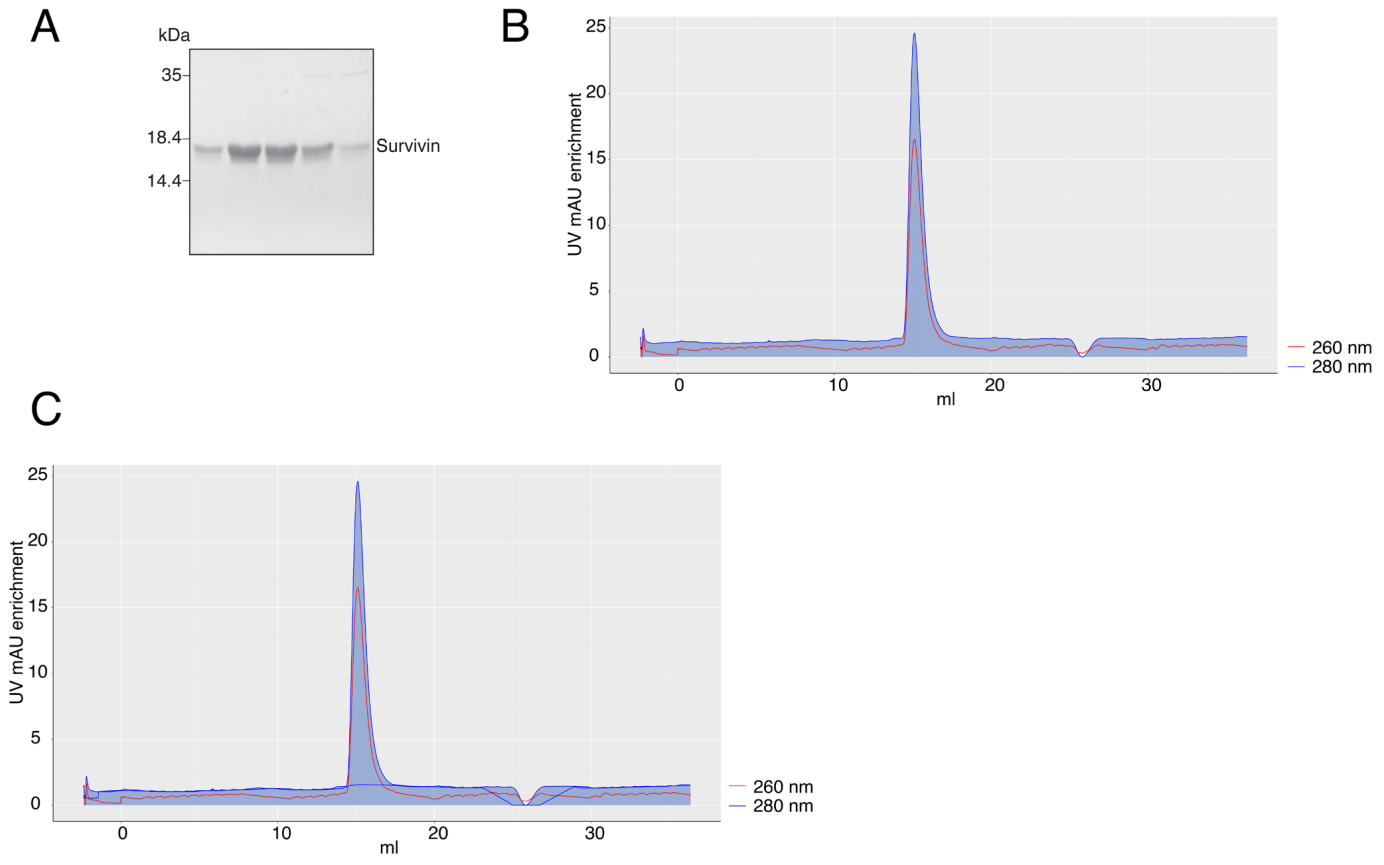
**A** – Coomassie-stained SDS-PAGE gel of purified Survivin. **B** – A chromatography plot with area highlighted under the 280 nm curve, exported from the app. **C** – A chromatography plot with area highlighted under the 280 nm curve and a baseline for the 280 nm curve, exported from the app. In **B** and **C**, the axis labels of the plots were modified in the image editor.

We purified Survivin ( Abad *et al.*, 2022; Abad *et al.*, 2019) ( Figure 1A), a member of the chromosomal passenger complex, which orchestrates cell division ( Adams *et al.*, 2000; Carmena *et al.*, 2012; Cooke *et al.*, 1987; Earnshaw & Bernat, 1991), and exported the .txt file from the Unicorn™ software after purification. Upon file upload, the app automatically plots normalized 260 nm and 280 nm mAU (milli-Absorbance Units) curves across the elution volume. The area under the curve is transparent by default, but can be filled with a user-defined color selected in the “Color” tab ( Figure 1B). The default curve colors, red for 260 nm and blue for 280 nm mAU, can also be changed in the same tab. A baseline of the same color as the curve can be fitted for the whole curve, but is most helpful under the peak of the purified protein ( Figure 1C). We note that although fitting a baseline is automated in Unicorn™, to our knowledge it is impossible to export it for plotting in a different software.

**Figure 2.**
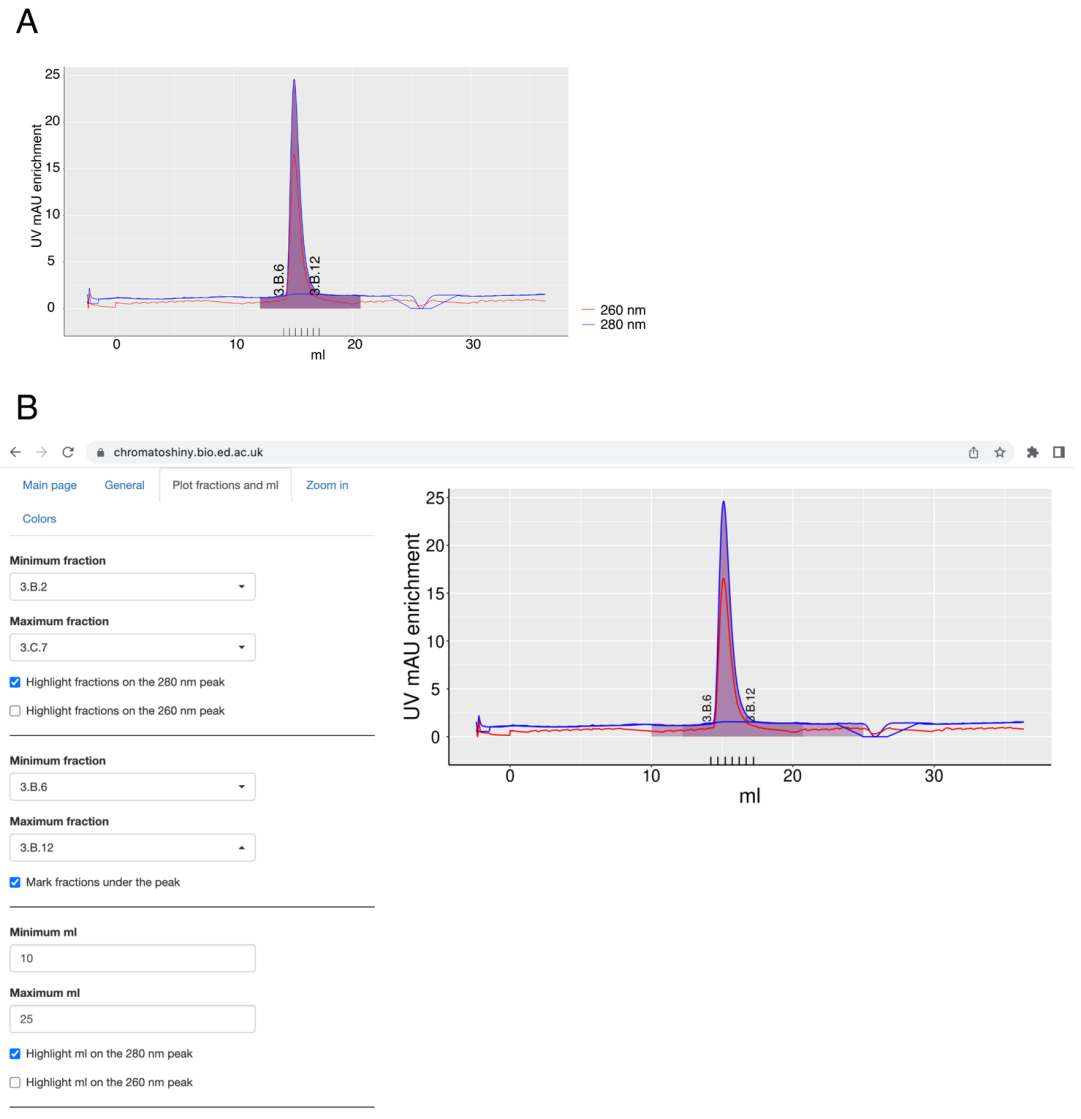
**A** – A chromatography plot with the areas of selected fractions highlighted under the curve and selected fractions under the plot plotted. The axis labels of the plot and the fractions labels were modified in the image editor. **B** – A screenshot from the app, where the plot was made with highlighted elution volume and selected fractions area under the curve. Plotted fractions under the curve are shown as well.

In the ChromatoShiny app, selected fractions or a particular elution volume can be plotted using the tab “Plot fractions and ml”. Upon file upload, a dropdown menu appears, from which the fractions to plot can be chosen. Fractions can be highlighted on the plot as an area under the curve (purple by default) and plotted under the plot ( Figure 2A). Thus, one can simultaneously highlight all fractions collected after elution and a subset of these fractions, e.g. those used for downstream experiments. Highlighting a defined elution volume as an area under the curve is also possible and not mutually exclusive with highlighting fractions ( Figure 2B). If the elution volume and the highlighted fractions are plotted at the same time, any overlay will be especially apparent if they are both plotted in partially transparent colors.

**Figure 3.**
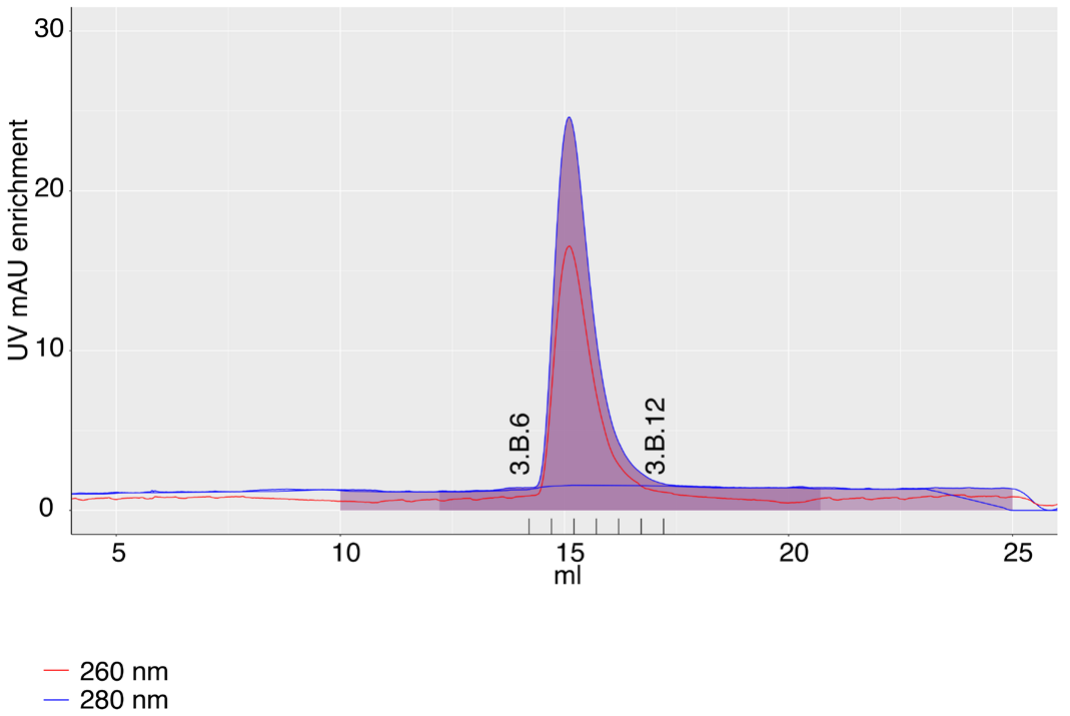
A chromatography plot from 2B with zoom in, exported from the app. The axis labels of the plot and the fractions labels were modified in the image editor.

During purifications, extra peaks often appear, which may have no relation to the eluting protein (e.g. contaminants). Furthermore, when several complexes are separated, one might want to zoom into areas with particular peaks. Survivin purification in the app can be plotted with "zoom in" into the Survivin peak after fitting the baseline, highlighting fractions and elution volume ( Figure 3). Zooming into the plot is fully compatible with all its other features described above.

Publication-quality figures can be saved through a “download” button. The plot can be exported either as a vector image in .pdf or .eps format (for further manipulation in image processing software without loss of resolution), or as a .tiff raster image.

## Discussion

Shiny apps are becoming more and more popular tools in the life sciences. To date, they range from analyzing data distribution in boxplots ( Spitzer *et al.*, 2014), to the analysis of sequencing data ( Knight *et al.*, 2021) and to the sorting and analysis of complex data sets ( Samejima *et al.*, 2022). Rather than taking screenshots from the Unicorn™ software after routine Äkta purifications, which is the most straightforward way to export the data, we decided to develop a Shiny app that would efficiently process files exported from Unicorn™ and give a publication quality plot as an output.

The Shiny app described here allows users to overlay several features of the chromatography plot outside the Unicorn™ software. The code developed for the app and posted on GitHub contains a subroutine for importing Unicorn™ .csv files, which can substitute for importing Unicorn™ .txt files. The function in the app working with the header of the imported table can be also slightly changed in the code and adapted to differing versions of the Unicorn™ software routinely used in different laboratories. The data from non-Äkta FPLC instruments can be entered in the template, available for downloading in the app and then saved as a .txt file and plotted accordingly. Furthermore, the app is well adapted for plotting many similar plots: upon new file upload, the features generated for the previous file are saved, with the exception of the fractions which are defined *de novo*.

The decision tree used in the main function of the app allows users to easily add and remove features from the plot and combine several features at a time. For example, the baseline can be plotted together with highlighting the fractions and elution volume, and it is possible to zoom in on any area of this modified plot.

We hope that the code provided for the app will help biochemistry and structural biology laboratories to build laboratory-specific pipelines for generation of high-quality publication plots after chromatographic purification on Äkta machines.

## Data Availability

Zenodo. ChromatoShiny: an interactive R/Shiny App for plotting chromatography profiles DOI. 10.5281/zenodo.10079398 This project contains the following underlying data: "011221_Run15_533.tif" - an uncropped gel scan (Fig. 1A) "CPC_SurvK62A_preps_CPC_SP_IE512.tif" - a scan of the marker used in the gel image in Fig. 1A (the gel on the right) "Fig. 1B.pdf" - a raw plot for Fig. 1B exported from the shiny app "Fig. 1C.pdf" - a raw plot for Fig. 1C exported from the shiny app "Fig. 2A.pdf" - a raw plot for Fig. 2A exported from the shiny app "Fig. 2B.png" - a raw screenshot from the shiny app for Fig. 2B "Fig. 3.pdf" - a raw plot for Fig. 3 exported from the shiny app Data are available under the terms of the Creative Commons Zero "No rights reserved" data waiver (CC0 1.0 Public domain dedication).

## Software availability

- source code available from: https://github.com/NatashaKochanova/ChromatoShiny-App
- Software available from: 10.5281/zenodo.10372578
- App accessible from: https://chromatoshiny.bio.ed.ac.uk/
- License: MIT license
