# Supplementary material for: ChromatoShiny: an interactive R/Shiny App for plotting chromatography profiles
Source: Wellcome Open Res. 2024 Jan 22;8:332. Originally published 2023 Aug 8. [Version 2] doi: 10.12688/wellcomeopenres.19708.2 (PMC10483177; doi:10.12688/wellcomeopenres.19708.2)
Supplement: Peer review history

# Reviewer report: Reviewer response for version 1

**DOI:** 10.21956/wellcomeopenres.21829.r65521

**Published:** 2023-09-06

**Authors:** Laura Spagnolo

**Affiliations:** 1 School of Molecular Biosciences, University of Glasgow, Glasgow, Scotland, UK

This manuscript describes a very handy new tool for the plotting and handling of chromatograms. ChromatoShiny will allow junior researchers a faster way of representing data, helping streamlining record keeping, data interpretation and representation in most biochemistry laboratories. In particular, this app promises a very intuitive route to clear cross-referencing chromatography profiles with data from SDS-PAGE, Western blot and activity assay analyses.

 This is a clearly written, technically sound report on a useful tool, which can be used by most researchers using chromatography in their experiments.

Are the conclusions about the tool and its performance adequately supported by the findings presented in the article?

Yes

Is the rationale for developing the new software tool clearly explained?

Yes

Is the description of the software tool technically sound?

Yes

Are sufficient details of the code, methods and analysis (if applicable) provided to allow replication of the software development and its use by others?

Yes

Is sufficient information provided to allow interpretation of the expected output datasets and any results generated using the tool?

Yes

Reviewer Expertise:

Structural biochemistry

I confirm that I have read this submission and believe that I have an appropriate level of expertise to confirm that it is of an acceptable scientific standard.

# Reviewer report: Reviewer response for version 1

**DOI:** 10.21956/wellcomeopenres.21829.r65520

**Published:** 2023-09-05

**Authors:** Sutapa Chakrabarti

**Affiliations:** 1 Institute of Chemistry and Biochemistry, Free University of Berlin, Berlin, Berlin, Germany

The ChromatoShiny app developed by Kochanova and co-authors promises to be a very helpful one for all biochemists that work with chromatography data generated by a semi-automated FPLC system such as the Äkta systems. Use of such data in a publication entails manually plotting the data using a software such as Microsoft Excel and then using a different graphics software such as Adobe Illustrator to label the graphs, as the graphs generated from the FPLC software are not of acceptable quality. For such users, the ChromatoShiny app would be immensely helpful. It has a number of interesting features that make it user-friendly a) an intuitive GUI b) no specialised format of input data c) options to pick traces to be plotted and baseline fit them d) zoom in and select areas of the chromatogram to be displayed as well as colours for the traces. 

 Undoubtedly, this app will be very useful and I imagine that even a fairly novice user will be able to use it without much support. I would have loved to try the app myself but it appears that the link is only for a "preview" version of the app and not ready for use as yet. 

 One minor point - it would be better if the term "Äkta liquid chromatography instruments" were to be replaced with FPLC instruments as there are other FPLC systems on the market, though understandably none as popular as the Äkta. Nevertheless, it broadens the use of the app by not restricting it to data generated by the Äkta purifiers alone. 

 For all the reasons detailed above, I recommend indexing of this article without further major changes.

Are the conclusions about the tool and its performance adequately supported by the findings presented in the article?

Yes

Is the rationale for developing the new software tool clearly explained?

Yes

Is the description of the software tool technically sound?

Yes

Are sufficient details of the code, methods and analysis (if applicable) provided to allow replication of the software development and its use by others?

Yes

Is sufficient information provided to allow interpretation of the expected output datasets and any results generated using the tool?

Yes

Reviewer Expertise:

Structural biochemistry on protein-RNA complexes involved in mRNA decay in eukaryotes.

I confirm that I have read this submission and believe that I have an appropriate level of expertise to confirm that it is of an acceptable scientific standard.
